# Assessing Quality of Life in Genetic Cardiomyopathies: A Scoping Review

**DOI:** 10.3390/ijerph23070833

**Published:** 2026-06-25

**Authors:** Lucrezia Tomberli, Fausto Barlocco, Annariina Koivu, Jari Hyttinen, Iacopo Olivotto, Enrica Ciucci

**Affiliations:** 1 Department of Education, Languages, Intercultures, Literatures and Psychology (FORLILPSI), University of Florence, 50121 Florence, Italy; enrica.ciucci@unifi.it; 2 Department of Experimental and Clinical Medicine, University of Florence, 50134 Florence, Italy; fausto.barlocco@unifi.it (F.B.); iacopo.olivotto@unifi.it (I.O.); 3 Faculty of Medicine and Health Technology, Tampere University, 33014 Tampere, Finland; annariina.koivu@tuni.fi (A.K.); jari.hyttinen@tuni.fi (J.H.)

**Keywords:** genetic cardiomyopathy, QoL measurements, psychosocial factors, patient-centred approaches, quality of life, health related quality of life, well-being

## Abstract

**Highlights:**

**Public health relevance—How does this work relate to a public health issue?**

- Genetic cardiomyopathies impose not only clinical but also psychosocial, functional, and economic burdens on patients and families.
- Mapping how Quality of Life (QoL) and Health-Related Quality of Life (HRQoL) are assessed can support more patient-centred approaches in chronic cardiovascular care.

**Public health significance—Why is this work of significance to public health?**

- This scoping review highlights the heterogeneity of QoL and HRQoL measurement tools used in genetic cardiomyopathy research.
- The findings show that psychosocial, social, and economic dimensions remain inconsistently assessed across studies.

**Public health implications—What are the key implications or messages for practitioners, policy makers and/or researchers in public health?**

- Greater conceptual clarity between QoL and HRQoL is needed to improve comparability across studies and clinical settings.
- Future research should integrate patient-reported outcomes, caregiver perspectives, and longitudinal designs to inform personalized and equitable care pathways.

**Abstract:**

Genetic cardiomyopathies (GCMs) are chronic heart muscle disorders requiring lifelong monitoring and treatment. Although quality of life (QoL) and health-related quality of life (HRQoL) are increasingly recognized as important outcomes in cardiomyopathy care, their conceptualization and measurement remain inconsistent. This scoping review aims to (a) identify the tools most commonly used to assess QoL and HRQoL in adults with genetic cardiomyopathies and (b) map the thematic areas of existing studies, including symptom burden, psychological distress, diagnostic challenges, and the impact of medical and psychological interventions. PubMed, Scopus, and PsycINFO were systematically searched, and the final search was completed in November 2025. Seventeen peer-reviewed studies met the inclusion criteria and were included in this scoping review. The review followed the PRISMA extension for Scoping Reviews and included both quantitative, qualitative and mixed-methods designs. Most studies employed standardized tools such as *EQ-5D* (N = 5), *SF-36*/SF36v2 (N = 5), and the *Kansas City Cardiomyopathy Questionnaire* (N = 3), while others included the *Minnesota Living with Heart Failure Questionnaire* (N = 2) and disease-specific or ad hoc measures. The most frequently investigated themes included impairments in physical functioning, emotional well-being, symptom burden, psychological distress, and social participation. Several studies showed that patients’ perceived QoL was more closely associated with symptom burden and psychological adjustment than with objective clinical indicators alone. Clinical interventions showed mixed or limited effects on QoL and HRQoL outcomes, even when clinical parameters improved. Qualitative research further emphasized the lived experiences of patients and families, highlighting unmet needs in care. Less commonly addressed findings concerned caregiver perspectives, patient–provider communication, treatment adherence, socioeconomic disadvantage, healthcare costs, productivity loss, and the experiences of patients with rarer cardiomyopathy-related conditions. The results highlight how QoL and HRQoL are central but still inconsistently assessed outcomes in cardiomyopathy research. This review calls for greater conceptual clarity between QoL and HRQoL, greater standardization in measurement tools, broader inclusion of psychosocial variables, and more patient-centred research approaches to better support individuals living with cardiomyopathies.

## 1. Introduction

Chronic illnesses represent a significant burden for adult patients, not only in terms of physical health but also in their psychological and social well-being [1,2,3]. Living with a chronic condition requires continuous medical management, lifestyle adjustments, and often long-term adherence to pharmacological treatments. These demands can lead to increased psychological distress, including anxiety, depression, and feelings of uncertainty about the future [4,5,6]. Furthermore, chronic illness can disrupt an individual’s sense of autonomy, self-efficacy, and social identity, affecting the overall perception of well-being [7,8,9,10,11].

One of the key constructs used to capture the multidimensional impact of chronic diseases is quality of life (QoL). The concept of QoL emerged within biomedical practice in the second half of the twentieth century, alongside the development of clinical trials, palliative care, and increasing sociological interest in patients’ subjective experiences of illness. Since then, it has become increasingly relevant in health research, despite the persistence of different definitions shaped by individual circumstances, cultural norms, and expectations [12].

QoL is a broad concept that encompasses individuals’ subjective perception of their well-being, integrating physical, psychological, and social dimensions [13,14,15,16]. While several definitions exist, the World Health Organization (WHO) defines QoL as individuals’ perception of their position in life within the context of their culture, values, goals, and concerns [17]. In medical and psychological research, health-related quality of life (HRQoL) is particularly relevant, as it focuses on how a person’s health condition affects their ability to lead a fulfilling life. HRQoL assessments typically include measures of physical functioning, emotional well-being, social participation, and disease-related burden [18,19,20]. Although QoL and HRQoL are closely related, they should not be considered fully interchangeable: QoL refers to a broader subjective appraisal of well-being, whereas HRQoL focuses more specifically on the impact of health status, symptoms, and treatment on daily life. In the cardiomyopathy literature, however, these terms are often used inconsistently or with partially overlapping meanings, making it important to map how they are operationalized across studies.

In the context of chronic diseases, numerous studies have explored how different medical conditions impact QoL. Research highlights that chronic illnesses often lead to a lower QoL due to the physical limitations they impose, as well as the associated emotional distress and social constraints [21,22,23]. For example, conditions such as diabetes, chronic respiratory diseases, and heart failure have been consistently linked to decreased QoL scores [24,25,26]. Understanding and measuring QoL in these populations is crucial for developing targeted interventions that improve patient well-being beyond clinical outcomes.

Genetic cardiomyopathies (GCMs) are a group of heart muscle disorders caused by genetic defects which can manifest at various ages and lead to progressive structural changes in hearts that generally appear normal at birth [27,28,29,30]. GCMs have a relevant epidemiological and clinical impact, although prevalence estimates vary according to disease subtype, diagnostic criteria, and diagnostic methods. Hypertrophic Cardiomyopathy (HCM) is commonly reported as the most frequent inherited cardiomyopathy, with prevalence estimates ranging from approximately 1:200 to 1:500 individuals [31], while the prevalence estimates of Dilated Cardiomyopathy (DCM) vary more widely, ranging from approximately 1:250 to 1:2500 individuals in the general population [32,33]. The recent guidelines by the *European Society of Cardiology* (ESC) also emphasize that the management of cardiomyopathies involves not only the affected individuals, but also their relatives, through family screening, genetic counselling, genetic testing, and long-term follow-up [34].

Unlike acquired cardiomyopathies, which develop later in life due to factors such as hypertension or ischemic heart disease, GCMs are present from early life and often require lifelong medical follow-up. They should not be confused with “congenital heart disease” characterized by anatomic malformations occurring during development in utero, which only rarely have a genetic basis.

These conditions can range from mild, asymptomatic forms to severe cases that lead to arrhythmias, heart failure, cardiac arrest, or the need for heart transplantation [35,36] and increased risk of sudden cardiac death [37]. The impact of GCMs extends beyond physical symptoms, influencing daily activities, emotional health, and long-term life planning. Adults with GCMs often experience fatigue, exercise intolerance, and limitations in their social and professional lives, all of which contribute to a reduced QoL [38,39]. Additionally, the unpredictability of the disease course can lead to heightened anxiety and concerns about disease progression [40,41,42].

Although QoL is increasingly recognized as a critical outcome in chronic disease management, the mapping of how QoL and HRQoL are assessed and conceptualized in adults with genetic cardiomyopathies remains an unmet need.

Understanding how to measure QoL effectively is essential for improving patient care and guiding clinical decision-making. This becomes particularly relevant in the present context of emerging precision cardiology, including initiatives such as projects for the development of patient-specific ‘digital twins’ (computational replicas of an individual heart, integrating clinical, genetic, and patient-reported data), which are opening new avenues for risk stratification, treatment personalization, and patient empowerment [43,44,45]. These innovations further underscore the need for a consistent, valid assessment of quality of life, enabling HRQoL to serve not only as a secondary outcome but also as a key input in personalized care pathways.

Exploring the key areas of interest within the literature, such as the impact of symptoms, diagnostic challenges, and the effects of medical and psychological interventions, can provide deeper insight into the lived experiences of individuals with these cardiac conditions. Given the complexity of genetic cardiomyopathies and their broad implications for well-being, a thorough examination of how QoL and HRQoL are assessed, operationalized, and thematically investigated is crucial for advancing both research and clinical practice. To date, the availability and consistency of data in this specific field remains unresolved. For this reason, a scoping review is particularly appropriate to map the existing evidence, identify the measurement tools used, clarify the main thematic areas addressed, and highlight gaps for future research.

## 2. The Present Study

*Quality of life* was specifically chosen as the focus of this investigation as an overarching construct that captures multiple dimensions of patients’ lived experience, including the physical, psychological, and social aspects of well-being. Given the partial overlap and inconsistent use of QoL and HRQoL in the literature on cardiomyopathies, both terms were considered in this review when referring to studies assessing the perceived impact of disease, symptoms, treatment, and daily life functioning.

The present scoping review aims to investigate (a) the tools available in the literature for assessing QoL and HRQoL in patients with genetic cardiomyopathies and (b) to explore which specific aspects of QoL and HRQoL have been most frequently examined in relation to cardiomyopathies, such as diagnostic challenges, symptom burden, the impact of medical and psychological interventions, and other key factors influencing patient well-being. By addressing these two objectives, this review aims to provide a more comprehensive understanding of both the methodologies used to measure QoL and HRQoL and the thematic focus of existing research, thereby identifying current gaps and informing future research directions.

## 3. Methods

### Search Strategy and Study Selection

The review was conducted and reported according to the PRISMA-ScR guidelines; the completed PRISMA 2020 Abstract Checklist and PRISMA 2020 Checklist are available in the Supplementary Materials (Tables S1 and S2).

**Figure 1:**
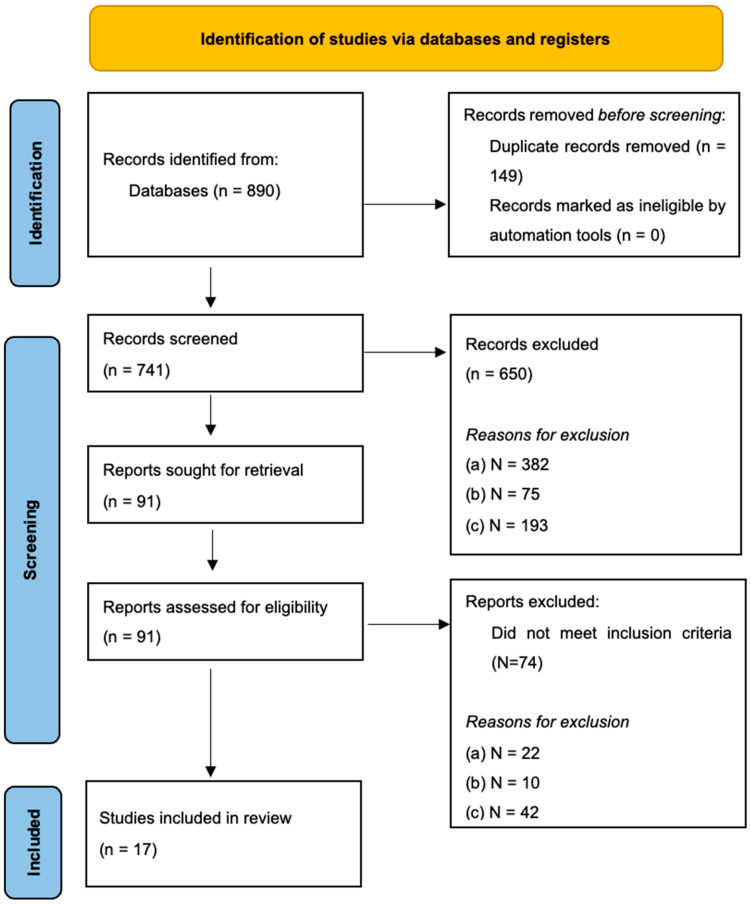
PRISMA flow diagram.

For this study, the screening and selection process was reported using a PRISMA flow diagram (Figure 1), in line with the PRISMA extension for Scoping Reviews (PRISMA-ScR):

Inclusion criteria: Studies were considered eligible if they met the following criteria:

(a) Focused on adult genetic cardiomyopathies;
(b) Examined the impact of GCMs on QoL and/or HRQoL of patients;
(c) Published in English in peer-reviewed international journals.

*Grey literature was excluded from the present review because the aim was to map peer-reviewed studies published in international scientific journals, ensuring consistency in the type of evidence considered.*

- Databases: The literature search was conducted in PubMed, Scopus, and PsycINFO and it was completed in November 2025. The search identified 890 records before duplicate removal: 311 from PubMed, 577 from Scopus, and 2 from PsycINFO.
- Keywords: The search query used was *“Genetic cardiomyopathy” AND “quality of life”*.

The protocol for this scoping review was not prospectively registered. Search strategy, eligibility criteria, and data extraction procedures were defined by the authors before study selection.

During the development of the search strategy, preliminary exploratory searches were conducted using alternative terms, including “health-related quality of life”, “HRQoL”, and “QoL”. However, because QoL and HRQoL are partially overlapping constructs and because terminology is not used consistently across the cardiomyopathy literature, the broader term “quality of life” was retained as the main search term. This choice was intended to maximize the sensitivity of the search and reduce the risk of excluding relevant studies that addressed HRQoL without using this exact abbreviation or terminology. A broader retrieval strategy was therefore combined with a more detailed screening process, during which studies referring to QoL, HRQoL, or health-related quality of life were considered eligible, provided that they met the remaining inclusion criteria.

Two authors independently screened the titles and abstracts of the retrieved records for inclusion. The full texts of potentially eligible articles were then assessed, and disagreements were resolved through discussion until consensus was reached. The screening process was therefore based on independent assessment followed by a deliberative consensus procedure, rather than on a reviewer-by-reviewer numerical coding matrix designed for inter-rater reliability analysis. Potentially eligible or uncertain records were discussed in depth, and full texts were assessed whenever eligibility could not be confidently determined from title and abstract alone. For this reason, Cohen’s kappa was not calculated, as independent pre-consensus decisions were not recorded in a numerical format suitable for agreement analysis. Inclusion and exclusion criteria were established in accordance with the study’s objectives. Grey literature was excluded to ensure a focus on the current state of peer-reviewed scientific research. Relevant data from the included studies (e.g., study design, sample characteristics, outcomes related to QoL or HRQoL) were extracted by two authors independently using a standardized form created in Zotero, and cross-checked for accuracy. Consistently with the aims of a scoping review, the extracted data were synthesized descriptively to map the available evidence, identify recurrent measurement tools, and summarize the main thematic areas addressed across studies.

## 4. Results

The database search initially identified 890 records. After removing duplicates, 741 records were screened by title and abstract. At this stage, 650 records were excluded because they clearly did not meet one primary eligibility criterion: (a) a lack of focus on adult GCMs, including studies addressing broader, acquired, or non-genetic cardiovascular conditions without extractable cardiomyopathy-specific data (n = 238), with other studies including pediatric samples (n = 144); (b) the absence of QoL, HRQoL, or related patient-reported outcomes (n = 75); (c) ineligible publication type, including reviews, guidelines, consensus statements, grey literature, or non-published material (n = 193). The remaining 91 reports were considered potentially eligible based on title and abstract screening and were therefore retrieved and assessed in full text.

Following full-text assessment, 74 reports were excluded because they did not meet the eligibility criteria upon detailed evaluation. Each excluded report was assigned to one primary reason for exclusion. A total of 17 studies were therefore included in the final scoping review (see Figure 1).

### 4.1. Overview of Included Studies

Table 1 provides a schematic overview of the descriptive characteristics of the 17 studies included in this work.

The studies were conducted in various countries, namely the Netherlands (n = 4) [50,53,58,61], USA (N = 4) [47,49,57,62], UK (n = 2) [51,59], Australia (n = 2) [54,60], Romania (n = 1) [48], Canada (n = 1) [46], Brazil (n = 1) [52], and Norway (N = 1) [55]. One study was conducted through an online survey, involving participants from various countries (n = 1) [56], specifically the UK, the USA, Canada, Australia, Germany, Sweden, Spain, India, Israel, Portugal, Greece, Cyprus, Czech Republic, Philippines, Sudan, Mauritius, Pakistan, Russia, Palestine, the Netherlands, Ukraine, and Austria.

**Figure 2:**
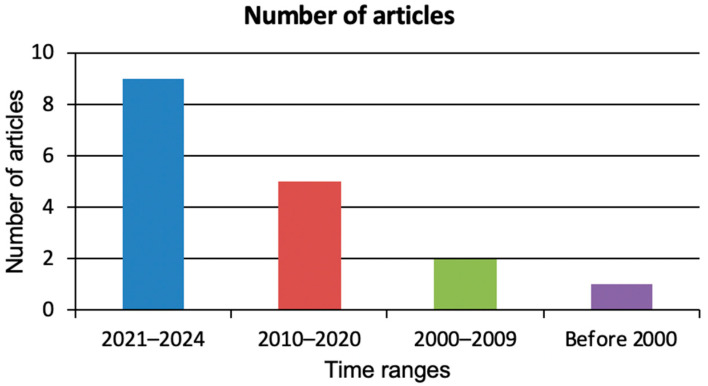
Temporal distribution of included studies.

Figure 2 illustrates the historical trend in the scientific literature on this topic.

Most of the studies were published between 2021 and 2024 (n = 9) [47,49,53,55,56,57,58,61,62] reflecting a growing interest in recent years (Figure 2). Five studies were also published between 2010 and 2020 [46,48,52,54,60], while fewer contributions were published between 2000 and 2009 [50,59].

Notably, only one study was published before 2000 (n = 1) [51].

All studies investigated patients’ perspectives, while only a few addressed the viewpoints of caregivers/partners or family members [47,57,62], or of physicians [62]. Sample sizes varied considerably across the included studies. Five studies involved very small groups of 7–16 participants [47,52,57,62], and one study included 40–50 patients [53]. Five studies included between 106 and 171 participants [48,51,55,59,60], and two studies included 228–486 patients [50,54]. Four larger studies reported samples of 500–789 patients [46,56,58,61], and one study included more than 1000 participants [49].

Overall, sample sizes ranged from very small groups of fewer than 20 participants with rare cardiomyopathies to large multicenter cohorts exceeding 1000 patients. This wide variability, together with the uneven distribution of cardiomyopathy subtypes, resulted in highly heterogeneous study populations.

Most of the studies (77%) employed standardized quantitative tools to assess QoL and/or HRQoL [46,48,49,52,53,54,55,58,59,60,61]. Two studies (12%) adopted a mixed-methods design, combining validated instruments with ad hoc or qualitative components [51,56]. Two studies (12%) relied exclusively on a qualitative approach, one using focus groups and semi-structured interviews with patients, caregivers, and clinicians [62], while Rintell and colleagues [57] explored psychosocial aspects of living with cardiomyopathy by employing semi-structured interviews rather than standardized QoL instruments. Therefore, it was also classified as qualitative.

The cardiomyopathies investigated in the included studies, based on the inclusion criteria of the present review, were as follows: hypertrophic cardiomyopathy—HCM (n = 4) [50,51,54,58], idiopathic dilated cardiomyopathy—DCM (n = 4) [48,53,59,61], ischemic cardiomyopathy (n = 2) [49,52], transthyretin amyloid cardiomyopathy—ATTR-CM (n = 2) [57,60], heart failure with preserved ejection fraction and subsequent cardiomyopathy (n = 1) [46]. Barth syndrome with associated cardiomyopathy (n = 1) [47]; limb-girdle muscular dystrophy R9—LGMDR9 with associated cardiomyopathy (N = 1) [55]; unspecified cardiomyopathy (n = 1) [56]; and long-chain fatty acid oxidation disorders associated with cardiomyopathy—LC-FAOD (n = 1) [62].

The studies included in this review appear heterogeneous in terms of sample size, study design, and perspectives considered. While most investigations focused on patients themselves, only a minority addressed the viewpoints of caregivers or clinicians. In addition, the distribution of cardiomyopathy subtypes and the publication years was uneven, with a concentration of contributions in the most recent decade. This heterogeneity supports the appropriateness of a scoping approach, as the available literature appears broad, methodologically diverse, and unevenly distributed across cardiomyopathy subtypes and patient populations.

### 4.2. Tools for Assessing Quality of Life in Patients with Genetic Cardiomyopathies

The analysis of the instruments used to assess QoL in the 17 included studies revealed a certain degree of methodological heterogeneity, although some tools were used recurrently.

The *EQ-5D* (*EuroQol-5 Dimensions*) questionnaire emerged as one of the most frequently employed instruments, having been used in five studies [49,53,58,60,61], specifically in its five-level version (EQ-5D-5L). This tool measures five key dimensions of health (mobility, self-care, usual activities, pain/discomfort, and anxiety/depression) and is particularly valued for its simplicity and the ability to derive a composite QoL and HRQoL index.

The *Short Form-36 Health Survey* (*SF-36*) was used in four studies [50,51,55,59]. An updated version, the SF-36v2, was used in the study by Ingles and colleagues [54]. These instruments assess eight domains of perceived health, covering both physical and mental components.

Two studies [48,52] employed the *Minnesota Living with Heart Failure Questionnaire* (*MLHFQ*), a disease-specific tool developed for patients with heart failure, designed to capture the impact of the condition on daily living.

The *Kansas City Cardiomyopathy Questionnaire* (*KCCQ*), specifically developed for patients with cardiomyopathies, was used in three studies [46,53,58], focusing on physical limitations, symptom burden, social functioning, and overall health perception.

Other specialized tools included the *Individualized Neuromuscular Quality of Life Questionnaire* (*INQoL*), used by Jensen and colleagues [55], and the *HCM Patient Experience Scale*, used by Ingles and colleagues [54] to assess subjective aspects of living with hypertrophic cardiomyopathy.

Two studies implemented ad hoc instruments: Cox and colleagues [51] supplemented the *SF-36* with a set of original scales exploring psychological adjustment, disease-related worries, involvement in care, and satisfaction with clinical relationships; Ormondroyd and colleagues [56], on the other hand, designed a 20-item survey addressing everyday experiences of living with cardiomyopathy, which was analyzed using both qualitative and quantitative methods.

Finally, one study [62] adopted an exclusively qualitative approach, using focus groups and semi-structured interviews with patients, caregivers, and clinicians involved in rare conditions such as long-chain fatty acid oxidation disorders (LC-FAODs).

Alongside assessment tools specifically directed at QoL and HRQoL, most studies also collected data on domains closely related to patients’ quality of life. These included the burden of physical symptoms, such as fatigue, dyspnea, or sleep disturbances; psychological well-being, often assessed through validated tools measuring anxiety, depression, or stress (e.g., the *Hospital Anxiety and Depression Scale*—HADS, the *Depression Anxiety Stress Scale*—DASS); and risk perception, explored through specific items assessing patients’ perceived vulnerability to disease progression or sudden cardiac events.

Several studies also addressed treatment adherence and self-management behaviours, employing tools such as the *Morisky Medication Adherence Scale* (*MMAS-8*), or ad hoc questions regarding the use of medications and healthcare services. Moreover, the patient–provider relationship and satisfaction with clinical care were investigated through instruments developed by the authors themselves, targeting aspects such as communication quality, trust in healthcare professionals, or perceived adequacy of information received during consultations. These instruments, while not directly measuring QoL or HRQoL, provide valuable contextual information that contributes to a broader understanding of the patient’s experience and the psychosocial impact of cardiomyopathies.

Across studies, both measurement use and reporting practices were heterogeneous, with several QoL domains underreported; overall, the available evidence base was limited and inconsistent, limiting cross-study comparability and any inference across cardiomyopathy subtypes. This finding is particularly relevant for a scoping review, as it highlights not only the range of tools currently used, but also the lack of conceptual and methodological alignment in how QoL and HRQoL are operationalized across studies.

Table 2 summerizes QoL measurement tools and study designs across the studies included in this scoping review.

### 4.3. Key Quality of Life Themes in Cardiomyopathy Research

The analyzed literature on cardiomyopathies and QoL or HRQoL revealed a growing interest in capturing the lived experiences of patients, not only through traditional clinical outcomes but also through psychosocial and economic lenses. Across the 17 studies reviewed, QoL and HRQoL were approached from multiple angles, reflecting the heterogeneity of cardiomyopathies and their impact on patients’ lives. Table 3 summerizes the aims of the included studies and the key results.

A prominent group of studies aimed to quantify the impact of genetic cardiomyopathies on HRQoL, often using validated questionnaires. These works assessed HRQoL impairments in patients with hypertrophic cardiomyopathy [48,51], dilated cardiomyopathy [59,61], or limb-girdle muscular dystrophy type R9 [55], highlighting limitations across physical, emotional, and social domains.

Several studies examined clinical interventions and their impact on QoL and HRQoL. For example, the therapeutic effects of *vericiguat*, a prescription medication used to treat chronic heart failure [46], CABG [49], gene therapy [52], and intravenous immunoglobulin [53] were investigated, revealing mixed outcomes—particularly limited benefit in HRQoL despite some clinical improvements. In contrast, interventions like cardiac devices were associated with improved perceptions of safety and better QoL in some groups [60,61].

Another key focus is the psychosocial and mental health burden experienced by patients and their families. High levels of anxiety and depression were common [47,51,59], with caregivers often reporting worse perceptions of patient well-being than patients themselves [47]. Notably, symptom severity and psychological adjustment were frequently stronger predictors of QoL than clinical severity alone [51,59].

The burden was further compounded by social and economic stressors [54], which were shown to negatively influence access to care and adherence to treatment.

Several studies explored genetic testing and risk perception, particularly among carriers of HCM mutations [50]. These findings suggested that the psychological impact of a genetic diagnosis can vary significantly depending on symptom status and perceived risk, highlighting the importance of personalized counselling and psychosocial support.

Qualitative and mixed method studies provided unique insights into patient experiences and preferences, particularly regarding the diagnostic journey [57], symptom perception [62], and attitudes towards genetic therapies [56]. These studies emphasized the need for multidisciplinary care and the development of patient-reported outcome measures that capture the complexity of living with cardiomyopathy.

Finally, population-level registries and burden-of-disease studies offered a broader perspective on the societal impact of cardiomyopathies. Large-scale registries enable comprehensive analyses of subtypes and prognostic factors, while economic evaluations [58,61] stressed the role of productivity loss and care costs, especially in symptomatic and working-age populations. In sum, the literature underscores the multidimensional nature of QoL and HRQoL in cardiomyopathy, shaped by medical, psychological, social, and economic factors. The findings support a shift towards more holistic, patient-centred approaches in both research and clinical care. At the same time, they also indicate that the conceptual boundaries between QoL, HRQoL, psychosocial adjustment, symptom burden, and lived experience are not always clearly defined across studies, reinforcing the need for greater clarity in future research.

## 5. Discussion

To our knowledge, this scoping review represents the first attempt to comprehensively map how QoL and HRQoL in patients with genetic cardiomyopathies have been assessed and conceptualized in the scientific literature. By examining both the instruments used and the specific domains explored, the present work offers a contribution at two different levels: a methodological overview of QoL and HRQoL measurement approaches to GCMs and a thematic synthesis of the most frequently investigated aspects of patient experience.

A key finding emerging from this review is the wide heterogeneity of instruments employed, reflecting a lack of standardization in QoL and HRQoL assessment across cardiomyopathy studies. Nonetheless, certain tools—such as the EQ-5D, the *Short Form Health Surveys* (SF-36, SF-12), and the *Kansas City Cardiomyopathy Questionnaire* (KCCQ)—have shown recurrent use, suggesting their perceived utility in capturing key dimensions of patient well-being. Disease-specific tools like the *MLHFQ* or the *HCM Patient Experience Scale* further demonstrate the growing effort to tailor QoL/HRQoL evaluation to specific patient populations. The integration of qualitative and ad hoc tools in a minority of studies also reflects an emerging attention to patient-reported experiences beyond standardized scales, particularly when addressing rare cardiomyopathies or underrepresented psychosocial domains. From a scoping review perspective, this heterogeneity should not be interpreted only as a methodological limitation, but also as an indicator of a field that is still developing its conceptual and measurement boundaries.

The frequent use of generic instruments such as the EQ-5D-5L and SF-36/SF-36v2, together with disease-specific tools such as the KCCQ and MLHFQ, also highlights the need to consider the different functions of these measurement approaches. Differences in the domains assessed, scoring systems, recall periods, and sensitivity to cardiomyopathy-specific symptoms may partly explain why different QoL/HRQoL tools can lead to heterogeneous or only partially comparable findings across studies. Generic instruments allow for comparisons across diseases, populations, and healthcare settings; in particular, the EQ-5D-5L can also be used to derive health utility values relevant for health economic evaluations. However, generic tools may be less sensitive to cardiomyopathy-specific symptoms, functional limitations, and treatment-related concerns. By contrast, disease-specific instruments such as the KCCQ or MLHFQ may better capture clinically meaningful changes in cardiac symptoms, physical limitations, and disease-related burden, but they are less suitable for comparisons across different conditions. Future studies on GCMs should therefore consider combining generic and disease-specific QoL/HRQoL measures to improve both comparability and clinical sensitivity.

Importantly, the literature reflects a shift in focus from purely clinical or survival outcomes to a more holistic understanding of disease burden. This shift is consistent with earlier and more recent work on QoL, HRQoL, and patient-perceived health status in cardiovascular disease. Foundational work by Kaplan [63] already emphasized that cardiovascular outcomes should not be evaluated only in terms of mortality or physiological indicators, but also in relation to patients’ perceived functioning and well-being. More recent reviews and meta-analyses in chronic cardiovascular conditions have similarly shown that QoL and HRQoL are shaped by physical limitations, symptom burden, emotional distress, social participation, and patients’ perceived ability to manage their condition [24,64].

Many studies included in this review underscore the discrepancy between objective measures of disease severity and patients’ perceived quality of life, highlighting the central role of psychological adjustment and individual coping mechanisms. This is also in line with previous evidence suggesting that patient-perceived health status is not only a patient-centred outcome but may also have prognostic relevance in chronic heart failure and coronary artery disease [65].

From a thematic perspective, the literature reviewed highlights several key research directions. Studies have addressed the impact of clinical interventions (e.g., pharmacological treatments, surgical procedures, gene therapy) on QoL, often revealing limited gains in well-being despite clinical improvements. This finding partly mirrors broader cardiovascular evidence showing that interventions may improve HRQoL, but that effects vary according to the type of intervention, population, and outcome measure used [66].

Others have explored the implications of genetic testing, patient–provider communication, and social determinants of health, all of which shape patient experiences in profound ways. Qualitative studies, in particular, emphasize the importance of lived experiences, offering rich narratives about diagnostic uncertainty, treatment burden, and family dynamics—elements that are often overlooked in large-scale clinical trials but that can be found in qualitative-based sociological and anthropological literature. Anthropological perspectives have further highlighted how cardiac technologies may be-come deeply embedded in patients’ identities and everyday lives, generating both feelings of safety and chronic psychological burden, as described in recipients of implantable car-dioverter defibrillators [67]. Compared with previous reviews on QoL and HRQoL in broader cardiovascular populations, the present scoping review adds a more specific focus on genetic cardiomyopathies. These conditions share several features with other chronic cardiovascular diseases, including symptom burden, functional limitation, emotional distress, and treatment impact. However, they also involve distinctive psychosocial and familial dimensions, such as inherited risk, genetic testing, implications for relatives, perceived risk of sudden cardiac death, and uncertainty about disease progression. These features may shape QoL and HRQoL in ways that are not fully captured by general cardiovascular QoL frameworks and support the need for more tailored assessment approaches in this population.

All these insights can contribute to developing the type of consistent, valid assessment of quality of life, and HRQoL in particular, to be used as a benchmark and goal for precision medicine interventions and personalized care pathways, including those based on various types of digital tools.

From a clinical perspective, this means that QoL and HRQoL assessment should not be considered only as a research outcome, but also as a routine component of cardiomyopathy care pathways. Brief, validated, patient-reported measures could be administered at key moments of care, including diagnosis, genetic counselling, treatment changes, device implantation, and follow-up visits. This would help clinicians identify persistent functional limitations, psychological distress, unmet informational needs, social difficulties, or family-related concerns, even when clinical parameters appear stable. Routine QoL and HRQoL monitoring may therefore support shared decision-making, timely referral to psychological or rehabilitation services, and more personalized, patient-centred management of genetic cardiomyopathies.

Despite the growing body of research, important gaps remain. There is limited consensus on the most appropriate QoL and HRQoL instruments for different subtypes of cardiomyopathy, and few studies have used longitudinal designs to track changes over time. The perspectives of caregivers and healthcare providers are underrepresented, economic aspects have only recently begun to be addressed, and comprehensive analyses of the societal costs associated with reduced QoL remain limited. Moreover, the literature appears unevenly distributed across cardiomyopathy subtypes, geographical contexts, and patient populations, with limited attention to equity-related variables such as socioeconomic status, gender, age, access to specialist care, and cultural differences in the experience and reporting of QoL.

Some limitations of the review process should be acknowledged. The search was limited to three databases and to peer-reviewed articles published in English, while grey literature was excluded. In addition, the search strategy was intentionally focused on genetic cardiomyopathies and quality of life, which may have excluded studies addressing related psychosocial outcomes without explicitly using QoL or HRQoL terminology. Similarly, the use of the overarching term “genetic cardiomyopathy” may have missed studies indexed primarily under specific cardiomyopathy subtypes, such as hypertrophic, dilated, arrhythmogenic, or restrictive cardiomyopathy. Future reviews focusing on individual cardiomyopathy subtypes may therefore benefit from broader subtype-specific search strings.

Finally, consistent with the aims of a scoping review, no formal risk of bias assessment or certainty-of-evidence assessment was conducted.

## 6. Conclusions and Future Directions

In conclusion, this review underscores the importance of assessing QoL and HRQoL as a central outcome in cardiomyopathy research and care. It advocates greater methodological consistency, increased use of patient-reported outcome measures, and expanded attention to psychological, social, and economic factors. Consistent with the aims of a scoping review, future research should first work toward clarifying which QoL and HRQoL domains are most relevant across different cardiomyopathy subtypes and identifying areas where current evidence remains fragmented or underdeveloped. More specifically, future studies should aim to (a) clarify the conceptual boundaries between QoL, HRQoL, psychosocial adjustment, symptom burden, and patient experience in this field; (b) identify a shared set of generic, disease-specific, and patient-reported measures that could improve comparability across studies; (c) integrate patients’, caregivers’, and clinicians’ perspectives to better capture the multidimensional burden of cardiomyopathies; (d) adopt longitudinal and mixed-methods designs to explore how QoL and HRQoL change across the disease trajectory; and (e) examine how QoL and HRQoL data may inform shared decision-making, genetic counselling, and personalized care pathways.

Further scoping work may also be useful to map QoL and HRQoL research within specific cardiomyopathy subtypes, rare genetic conditions, and underrepresented populations, as well as to examine whether existing instruments adequately capture culturally, socially, and economically diverse experiences of living with these conditions.

Ultimately, improving QoL for individuals living with cardiomyopathies requires not only advances in clinical management but also a deeper understanding of what it means to live with this complex and multifaceted group of conditions.

## Figures and Tables

**Table 1:** Characteristics of Included Studies.

Articles	Keywords	Country Where the Study Was Conducted	Journal	Target
Armstrong et al. (2020) [46]	/	Canada	Jama	789 patients
Bath et al. (2022) [47]	Barth syndrome; cardiomyopathy; quality of life; anxiety; depression; genetics; psychosocial	USA	*Journal of Cardiovascular Development and Disease*	16 patients and 10 caregivers
Capota et al. (2020) [48]	Hypertrophic cardiomyopathy, Quality of life, Heart failure, Kansas city cardiomyopathy questionnaire	Romania	*Health and Quality of Life Outcomes*	110 patients
Chew et al. (2022) [49]	Cardiomyopathies, coronary artery bypass, coronary artery disease, cost–benefit analysis, costs and cost analysis	USA	*Circulation*	1212 patients
Christiaans et al. (2009) [50]	Hypertrophic cardiomyopathy; quality of life; psychological distress; genetic testing	Netherlands	*American Journal of Medical Genetics*	228 patients
Cox et al. (1997) [51]	hypertrophic cardiomyopathy; quality of life; patient satisfaction	UK	*Heart*	171 patients
Giusti et al. (2013) [52]	/	Brazil	*Human Gene Therapy Methods*	13 patients
Hazebroek et al. (2021) [53]	Dilated cardiomyopathy, Heart failure, Intravenous immunoglobulin, Parvovirus B19, Endomyocardial biopsy	Netherlands	*European Journal of Heart Failure*	50 patients
Ingles et al. (2015) [54]	Hypertrophic cardiomyopathy Social determinants of health Adherence	Australia	*International Journal of Cardiology*	486 patients
Jensen et al. (2024) [55]	Muscular dystrophies, Limb-girdle, quality of life, sleep quality, fatigue	Norway	*Journal of Neuromuscular Diseases*	135 patients
Ormondroyd et al. (2024) [56]	/	UK, USA, Canada, Australia, Germany, Sweden, Spain, India, Israel, Portugal, Cyprus, Greece, Czech Republic, Philippines, Mauritius, South Sudan, Pakistan, Russian federation, Palestine, Netherlands, Ukraine, Austria	*European Journal of Human Genetics*	634 patients
Rintell et al. (2021) [57]	/	USA	*Orhanet Journal of Rare Diseases*	17 patients and 5 caregivers
Schoonvelde et al. (2024) [58]	/	Netherlands	*European Heart Journal—Quality of Care and Clinical Outcomes*	506 patients
Steptoe et al. (2000) [59]	Dilated cardiomyopathy; quality of life; adjustment; well-being	UK	*Heart*	106 patients
Sweeting et al. (2017) [60]	Exercise, lifestyle, implantable cardioverter defibrillator, inherited heart disease	Australia	*European Journal of Cardiovascular Nursing*	155 patients
Wiethoff et al. (2024) [61]	Burden of disease, Dilated cardiomyopathy, Healthcare resource utilization, Societal costs, Quality of life	Netherlands	*European Heart Journal—Quality of Care and Clinical Outcomes*	550 patients
Williams-Hall et al. (2022) [62]	Long-chain fatty acid oxidation disorders, multi-method, multi-perspective, patient experience, qualitative, quality of life, symptoms	USA	*Therapeutic Advances in Endocrinology and Metabolism*	8 participants in the focus group (4 patients and 4 caregivers); 5 patients and 4 clinicians for the individual interviews

**Table 2:** QoL Measurement Tools and Study Designs Across 17 Included Studies.

Citation	Study Design	QoL Instruments	Other Instruments
Armstrong et al., 2020 [46]	Quantitative	KCCQ-PLS	6-Minute Walk Distance (6MWD)
Bath et al., 2022 [47]	Quantitative	PedsQL (pediatric QoL), PROMIS Short Form 8A (anxiety/depression, caregivers)	PROMIS Short Form 8A (Anxiety and Depression)
Capota et al., 2020 [48]	Quantitative	MLHFQ	NYHA class, eGFR, echocardiography, comorbidities
Chew et al., 2022 [49]	Quantitative	EQ-5D 3-level	None specified
Christiaans et al., 2009 [50]	Quantitative	SF-36	HADS, IPQ-R, Risk Perception, Satisfaction Inventory
Cox et al., 1997 [51]	Mixed methods	SF-36; HADS; ad hoc scales (Adjustment to HCM, Worry, Involvement, Patient Satisfaction)	HADS, Adjustment to HCM Scale, Worry Scale, Involvement Scale, Patient Satisfaction Scale
Giusti et al., 2013 [52]	Quantitative	MLHFQ	NYHA class, CCS classification, treadmill test, oxygen consumption, SPECT
Hazebroek et al., 2021 [53]	Quantitative	EQ-5D-5L; KCCQ	NYHA class, LVEF, Echocardiography, Clinical variables
Ingles et al., 2015 [54]	Quantitative	SF-36v2, HADS, HCM Experience Scale	MMAS-8, Demographics
Jensen et al., 2024 [55]	Quantitative	INQoL, SF-36	PSQI, FSS
Ormondroyd et al., 2024 [56]	Mixed methods	Ad hoc survey (20 cardiomyopathy items; qualitative and quantitative analysis)	None
Rintell et al., 2021 [57]	Qualitative	Semi-structured interviews with patients and caregivers (no standardized QoL instruments)	DASS, FPAS, FSAS
Schoonvelde et al., 2024 [58]	Quantitative	EQ-5D-5L; KCCQ	None reported
Steptoe et al., 2000 [59]	Quantitative	SF-36	HADS, IPQ-R, Risk Perception Scale, Satisfaction Scales
Sweeting et al., 2017 [60]	Quantitative	EQ-5D-5L	None specified
Wiethoff et al., 2024 [61]	Quantitative	EQ-5D-5L	iMCQ, iPCQ, DBC codes for in-hospital care
Williams-Hall et al., 2022 [62]	Qualitative	Focus groups and semi-structured interviews with patients, caregivers, clinicians	Focus groups and interviews with patients, caregivers, clinicians

**Table 3:** Summary of Study Objectives and Key Results.

Article	Aim of the Study	Results Summary
Armstrong et al., (2020) [46]	To evaluate the efficacy of the soluble guanylate cyclase stimulator vericiguat on the Physical Limitation Score (PLS) of the Kansas City Cardiomyopathy Questionnaire (KCCQ).	Vericiguat did not significantly improve KCCQ-PLS or 6MWD in HFpEF patients, suggesting limited HRQoL benefit in this population.
Bath et al., (2022) [47]	To explore the impact of living with this syndrome, associated with cardiomyopathy, on patients’ quality of life and overall well-being.	Patient- and caregiver-reported QoL differed, with caregivers generally reporting worse outcomes. Findings supported long-term QoL monitoring and psychosocial screening in clinical care.
Capota et al. (2020) [48]	This study evaluated the negative impact of heart failure (HF) on HRQoL in patients with hypertrophic cardiomyopathy (HCM), and investigated the main clinical determinants associated with HRQoL deterioration in this population.	Patients with HCM and HF showed moderate HRQoL impairment. Worse HRQoL was associated with older age, higher NYHA class, renal dysfunction, pulmonary hypertension, female sex, and structural cardiac abnormalities.
Chew et al. (2022) [49]	To estimate the lifetime costs and benefits of coronary artery bypass grafting (CABG) versus medical therapy (MED), based on patient-level resource utilization and clinical outcomes from the STICH trial.	CABG showed higher initial costs than medical therapy but greater long-term value through increased life expectancy and QoL, especially in severe ischemic cardiomyopathy.
Christiaans et al. (2009) [50]	To assess long-term QoL and psychological distress in HCM mutation carriers, with and without symptoms, compared to normative population data; to evaluate the impact of predictive DNA testing; and to identify factors associated with poorer QoL and psychological distress, including sociodemographic, clinical, and illness perception…	Mutation status alone did not reduce well-being. Lower HRQoL was mainly associated with manifest HCM, symptoms, perceived SCD risk, and physical comorbidities, rather than predictive DNA testing itself.
Cox et al. (1997) [51]	To evaluate HRQoL and psychological well-being in patients with hypertrophic cardiomyopathy, and to examine their associations with clinical symptoms and psychosocial factors.	Patients with HCM reported substantial physical, social, and psychological limitations. QoL was more strongly linked to symptoms and psychological adjustment than to objective clinical measures.
Giusti et al. (2013) [52]	To evaluate the safety, feasibility, and clinical and perfusion outcomes of gene therapy in patients with refractory angina.	VEGF165 gene therapy was feasible and safe, with temporary perfusion benefits and sustained improvements in symptoms, functional capacity, and QoL over one year.
Hazebroek et al. (2021) [53]	To investigate the added benefits of intravenous immunoglobulin (IVIg) over standard therapy in patients with idiopathic chronic dilated cardiomyopathy (DCM) and persistent parvovirus B19 infection.	IVIg showed no benefit on cardiac function, functional capacity, or QoL in chronic idiopathic DCM with persistent parvovirus B19.
Ingles et al. (2015) [54]	To examine the socioeconomic profile of patients attending a multidisciplinary clinic and assess its impact on clinical outcomes, psychosocial well-being, and adherence to treatment.	Socioeconomically disadvantaged patients were underrepresented in specialist care and showed poorer psychological health, disease understanding, QoL, and clinical profiles.
Jensen et al. (2024) [55]	The study examined changes in HRQoL over 14 months in a Norwegian LGMDR9 cohort, identifying priority health domains, high-risk subgroups, and key patient-reported symptoms. It also assessed the prevalence and correlates of fatigue and poor sleep quality.	Patients showed physical, emotional, and social HRQoL impairments. Fatigue and weakness predicted disease burden, with worse outcomes among females and homozygous patients.
Ormondroyd et al. (2024) [56]	The study explored patient and family perspectives on living with cardiomyopathy and their attitudes toward specific aspects of genetic therapies.	Patients and families showed strong support for gene therapy development, alongside concerns about future risks, family impact, counselling, communication, and equitable access.
Rintell et al. (2021) [57]	The study investigated the diagnostic journey, symptom burden, and QoL impact experienced by individuals with rare and frequently misdiagnosed conditions.	Patients and caregivers with ATTR reported delayed diagnosis, limited specialist care, and substantial emotional and physical burden, supporting the need for timely diagnosis and family-centred support.
Schoonvelde et al. (2024) [58]	The study provided both generic and disease-specific data on QoL and examined the healthcare and societal costs of HCM in a multicenter Dutch cohort, adopting a holistic, patient-centred perspective.	Asymptomatic HCM carriers showed QoL comparable to the general population, whereas symptomatic, younger, female, and obstructive HCM patients reported lower QoL and greater costs.
Steptoe et al. (2000) [59]	The study assessed HRQoL and psychological well-being in patients with dilated cardiomyopathy, examining their associations with clinical variables and psychological adjustment.	Patients with DCM showed severe limitations in physical functioning, social relationships, energy, sleep, and mental health. Psychological adjustment independently predicted QoL and distress.
Sweeting et al. (2017) [60]	The study examined the impact of implantable cardioverter defibrillators (ICDs) on physical activity levels and identified factors associated with activity engagement.	ICDs did not reduce physical activity, but about half of patients did not meet exercise recommendations. Shock-related anxiety limited activity, while device confidence supported engagement.
Wiethoff et al. (2024) [61]	The study assessed QoL and societal costs in patients with dilated cardiomyopathy (DCM).	Over 40% of patients with DCM reported mobility, pain, and daily activity limitations. Costs and QoL impairment increased with disease severity, mainly driven by productivity losses.
Williams-Hall et al. (2022) [62]	The study aimed to explore patients’ experiences, focusing on symptom perception and its impact on quality of life.	Long-chain fatty acid oxidation disorders (LC-FAOD) caused substantial lifestyle limitations and emotional burden, with episodic crises affecting social life, schooling, and work. Patient experience data highlighted unmet treatment needs.

## Data Availability

No new data were created or analyzed in this study. All data are available from the original sources cited in the manuscript.

## Supplementary Materials

The following supporting information can be downloaded at: https://www.mdpi.com/article/10.3390/ijerph23070833/s1, Table S1: PRISMA 2020 for Abstract Checklist; Table S2: PRISMA 2020 Checklist. Ref. [68] was cited in Supplementary Materials.
