# Supplementary material for: Assessing Quality of Life in Genetic Cardiomyopathies: A Scoping Review
Source: Int J Environ Res Public Health. 2026 Jun 25;23(7):833. doi: 10.3390/ijerph23070833 (PMC13410338; doi:10.3390/ijerph23070833)
Supplement: Supplementary file 1 [file ijerph-23-00833-s001.zip › ijerph-4345414-Table S2 PRISMA_2020_checklist.pdf]

PRISMA

PRISMA 2020 Checklist

Section and Topic	Item #	Checklist item	Location where item is reported
**TITLE**			
Title	1	Identify the report as a systematic review.	Title page. The title identifies the manuscript as a scoping review.
**ABSTRACT**			
Abstract	2	See the PRISMA 2020 for Abstracts checklist.	Abstract.
**INTRODUCTION**			
Rationale	3	Describe the rationale for the review in the context of existing knowledge.	Introduction, p. 2-6
Objectives	4	Provide an explicit statement of the objective(s) or question(s) the review addresses.	The Present Study section.
**METHODS**			
Eligibility criteria	5	Specify the inclusion and exclusion criteria for the review and how studies were grouped for the syntheses.	Methods, Search strategy and study selection section.
Information sources	6	Specify all databases, registers, websites, organisations, reference lists and other sources searched or consulted to identify studies. Specify the date when each source was last searched or consulted.	Methods, Search strategy and study selection section. PubMed, Scopus, and PsycINFO were searched; the search was completed in November 2025.
Search strategy	7	Present the full search strategies for all databases, registers and websites, including any filters and limits used.	Methods, Search strategy and study selection section;
Selection process	8	Specify the methods used to decide whether a study met the inclusion criteria of the review, including how many reviewers screened each record and each report retrieved, whether they worked independently, and if applicable, details of automation tools used in the process.	Methods, Search strategy and study selection section. Two authors independently screened titles/abstracts and full texts; disagreements were resolved by discussion until consensus was reached.
Data collection process	9	Specify the methods used to collect data from reports, including how many reviewers collected data from each report, whether they worked independently, any processes for obtaining or confirming data from study investigators, and if applicable, details of automation tools used in the process.	Methods, Search strategy and study selection section. Data were independently extracted by two authors using a standardized form and cross-checked for accuracy.
Data items	10a	List and define all outcomes for which data were sought. Specify whether all results that were compatible with each outcome domain in each study were sought (e.g. for all measures, time points, analyses), and if not, the methods used to decide which results to collect.	Methods, Search strategy and study selection section. Data were extracted on QoL/HRQoL outcomes, QoL assessment tools, symptom burden, psychological distress, diagnostic issues, interventions, and psychosocial aspects.
	10b	List and define all other variables for which data were sought (e.g. participant and intervention characteristics, funding sources). Describe any assumptions made about any missing or unclear information.	Methods, Search strategy and study selection section; Results, Overview of included studies.

PRISMA

PRISMA 2020 Checklist

Section and Topic	Item #	Checklist item	Location where item is reported
			Extracted variables included study design, country, sample characteristics, cardiomyopathy subtype, target population, instruments used, and main findings.
Study risk of bias assessment	11	Specify the methods used to assess risk of bias in the included studies, including details of the tool(s) used, how many reviewers assessed each study and whether they worked independently, and if applicable, details of automation tools used in the process.	Not applicable. A formal risk of bias assessment was not conducted, as this was a scoping review aimed at mapping the available evidence rather than estimating intervention effects or producing pooled effect estimates.
Effect measures	12	Specify for each outcome the effect measure(s) (e.g. risk ratio, mean difference) used in the synthesis or presentation of results.	Not applicable. No meta-analysis or quantitative effect synthesis was conducted.
Synthesis methods	13a	Describe the processes used to decide which studies were eligible for each synthesis (e.g. tabulating the study intervention characteristics and comparing against the planned groups for each synthesis (item #5)).	Methods, Search strategy and study selection section. All included studies were synthesized descriptively according to study characteristics, QoL/HRQoL measurement tools, and thematic areas.
	13b	Describe any methods required to prepare the data for presentation or synthesis, such as handling of missing summary statistics, or data conversions.	Methods, Search strategy and study selection section. Extracted data were organized in descriptive tables; no statistical transformation or conversion was performed.
	13c	Describe any methods used to tabulate or visually display results of individual studies and syntheses.	Results, Tables 1–3 and Figures 1–2.
	13d	Describe any methods used to synthesize results and provide a rationale for the choice(s). If meta-analysis was performed, describe the model(s), method(s) to identify the presence and extent of statistical heterogeneity, and software package(s) used.	Methods, Search strategy and study selection section. A descriptive synthesis was conducted, consistent with the aims of a scoping review.
	13e	Describe any methods used to explore possible causes of heterogeneity among study results (e.g. subgroup analysis, meta-regression).	Not applicable. No subgroup analysis, meta-regression, or statistical exploration of heterogeneity was conducted. Heterogeneity was addressed descriptively in the Results and Discussion sections.
	13f	Describe any sensitivity analyses conducted to assess robustness of the synthesized results.	Not applicable. No sensitivity analyses were conducted because

PRISMA

PRISMA 2020 Checklist

Section and Topic	Item #	Checklist item	Location where item is reported
			no meta-analysis or pooled quantitative synthesis was performed.
Reporting bias assessment	14	Describe any methods used to assess risk of bias due to missing results in a synthesis (arising from reporting biases).	Not applicable. Formal assessment of reporting bias was not conducted, as this scoping review did not include meta-analysis or outcome-level effect synthesis.
Certainty assessment	15	Describe any methods used to assess certainty (or confidence) in the body of evidence for an outcome.	Not applicable. Certainty of evidence was not formally assessed, as the review aimed to map the extent, characteristics, and thematic focus of the literature.
**RESULTS**			
Study selection	16a	Describe the results of the search and selection process, from the number of records identified in the search to the number of studies included in the review, ideally using a flow diagram.	Results, Study selection section; Figure 1, PRISMA flow diagram.
	16b	Cite studies that might appear to meet the inclusion criteria, but which were excluded, and explain why they were excluded.	Results, Study selection section; Figure 1, PRISMA flow diagram. Reasons for full-text exclusion are reported in the flow diagram.
Study characteristics	17	Cite each included study and present its characteristics.	Results, Overview of included studies; Table 1.
Risk of bias in studies	18	Present assessments of risk of bias for each included study.	Not applicable. A formal risk of bias assessment was not conducted for the reasons stated under Item 11.
Results of individual studies	19	For all outcomes, present, for each study: (a) summary statistics for each group (where appropriate) and (b) an effect estimate and its precision (e.g. confidence/credible interval), ideally using structured tables or plots.	Results, Tables 1–3.
Results of syntheses	20a	For each synthesis, briefly summarise the characteristics and risk of bias among contributing studies.	Results, Overview of included studies; Tools for Assessing Quality of Life; Key Quality of Life Themes in Cardiomyopathy Research. Risk of bias was not formally assessed.
	20b	Present results of all statistical syntheses conducted. If meta-analysis was done, present for each the summary estimate and its precision (e.g. confidence/credible interval) and measures of statistical heterogeneity. If comparing groups, describe the direction of the effect.	Not applicable. No meta-analysis or statistical synthesis was conducted.
	20c	Present results of all investigations of possible causes of heterogeneity among study results.	Not applicable. No statistical investigation of heterogeneity was conducted. Heterogeneity is discussed descriptively in the Results and Discussion sections.
	20d	Present results of all sensitivity analyses conducted to assess the robustness of the synthesized results.	Not applicable. No sensitivity

Section and Topic	Item #	Checklist item	Location where item is reported
			analyses were conducted.
Reporting biases	21	Present assessments of risk of bias due to missing results (arising from reporting biases) for each synthesis assessed.	Not applicable. Reporting bias was not formally assessed.
Certainty of evidence	22	Present assessments of certainty (or confidence) in the body of evidence for each outcome assessed.	Not applicable. Certainty of evidence was not formally assessed.
**DISCUSSION**			
Discussion	23a	Provide a general interpretation of the results in the context of other evidence.	Discussion, paragraphs 1–5.
	23b	Discuss any limitations of the evidence included in the review.	Discussion, final paragraphs; Conclusions and Future Directions.
	23c	Discuss any limitations of the review processes used.	Discussion, limitations paragraph.
	23d	Discuss implications of the results for practice, policy, and future research.	Discussion; Conclusions and Future Directions.
**OTHER INFORMATION**			
Registration and protocol	24a	Provide registration information for the review, including register name and registration number, or state that the review was not registered.	Methods, Search strategy and study selection section. The review was not prospectively registered.
	24b	Indicate where the review protocol can be accessed, or state that a protocol was not prepared.	Methods, Search strategy and study selection section. A protocol was developed a priori by the authors but was not prospectively registered or publicly available.
	24c	Describe and explain any amendments to information provided at registration or in the protocol.	Not applicable. No amendments to a registered or publicly available protocol were made.
Support	25	Describe sources of financial or non-financial support for the review, and the role of the funders or sponsors in the review.	Funding Statement.
Competing interests	26	Declare any competing interests of review authors.	Conflict of Interest Statement.
Availability of data, code and other materials	27	Report which of the following are publicly available and where they can be found: template data collection forms; data extracted from included studies; data used for all analyses; analytic code; any other materials used in the review.	Data Availability Statement.

From: Page MJ, McKenzie JE, Bossuyt PM, Boutron I, Hoffmann TC, Mulrow CD, et al. The PRISMA 2020 statement: an updated guideline for reporting systematic reviews. BMJ 2021;372:n71. doi: 10.1136/bmj.n71.
This work is licensed under CC BY 4.0. To view a copy of this license, visit https://creativecommons.org/licenses/by/4.0/
